# Health related quality of life in untreated and treated patients with AIS. Study II: the impact of treatment

**DOI:** 10.1186/1748-7161-9-S1-O77

**Published:** 2014-12-04

**Authors:** Manuel Rigo, Monica Villagrasa, Elisabetta DAgata

**Affiliations:** 1 Elena Salva Institut, Barcelona, Spain; 2 Institut Reçerca Vall Hebron, Barcelona, Spain

## Background

The relationship between scoliosis magnitude, HRQL and Self Perception of Trunk Deformity is not clear. To better understand this, a prospective data collection including age, main thoracic and lumbar or thoracolumbar Cob angle, Trunk Asymmetry Perception Scale TAPS (1), SRS-22 (2,3) and current treatment was started involving all patients with idiopathic scoliosis attending a rehabilitation clinic, 10 years of age or older at the time of consultation. The data have been retrospectively analyzed and are presented in several studies. This is the study II.

## Purpose

The aim of this study was to determine whether patients showed differences in their HRQL depending on the type of treatment.

## Methods

N 240; mean age 19.3 y + 10.3 (10-62), mean Cobb thoracic 33.7º + 13.8, lumbar or thoracolumbar 29.7 + 12.7. At the time of consultation patients were or had been untreated or treated (exercises exclusively, Chêneau brace + Scoliosis Physiotherapy Exercises, other braces, surgery). Statistics: SPSS.

## Results

Untreated patients: N 76; Mean Cobb Thoracic 19º; Lumbar/TL 17.5º; Means SRS-22 Pain 21.07; SRS-22 MH 19.64; SRS-22 SI 18.66; SRS-22 F 22.38; SRS-22 Sub Total 81.83. Treated with Exercises exclusively: N 36; Thoracic 24.6º; Lumbar/TL 22.4º; Pain 21.19; MH 20.11; SI 18.94; F 21.75; Sub Total 82.03. Treated with Chêneau+SPE: N 56; Thoracic 29º; Lumbar/TL 22.2º; Pain 22.75; MH 20.20; SI 19.55; F 22.71; Sub Total 85.25. Treated with other braces: N 69; Thoracic 34º; Lumbar/TL 24º; Pain 22.07; MH 20.06; SI 18.25; F 22.65; Sub Total 83.03.

No significant differences between the untreated group, exercises and other braces. Chêneau+SPE showed better scores in Pain and Function, with bigger curves, in comparison with untreated and treated only with exercises and in SI, with lower curves, in comparison with those treated with other braces.

## Discussion and conclusions

This type of study does not allow to drawn final conclusions but it shows that HRQL seems to be similar in untreated (with milder curves) and treated patients (with bigger curves), all attending a rehabilitation clinic for medical advice or for control, suggesting some positive treatment impact.

